# Mid-infrared absorption by soft tissue sarcoma and cell ablation utilizing a mid-infrared interband cascade laser

**DOI:** 10.1117/1.JBO.26.4.043012

**Published:** 2021-04-21

**Authors:** Eric Larson, Madeline Hines, Munir Tanas, Benjamin Miller, Mitchell Coleman, Fatima Toor

**Affiliations:** a University of Iowa, Electrical and Computer Engineering Department, Iowa City, Iowa, United States; b University of Iowa Hospitals and Clinics, Department of Radiation Oncology, Iowa City, Iowa, United States; c University of Iowa Hospitals and Clinics, Department of Pathology, Iowa City, Iowa, United States; d University of Iowa Hospitals and Clinics, Department of Orthopedics and Rehabilitation, Iowa City, Iowa, United States; e University of Iowa Hospitals and Clinics, Holden Comprehensive Cancer Center, Experimental Therapeutics Program, Iowa City, Iowa, United States

**Keywords:** infrared spectroscopy, mid-infrared, soft-tissue sarcoma, interband cascade laser, cancer cell ablation

## Abstract

**Significance:** Mid-infrared (MIR) light refers to wavelengths ranging from 3 to 30  μm and is the most attractive spectral region for ablation of soft and hard tissues. This is because building blocks of biological tissue, such as water, proteins, and lipids, exhibit molecular vibrational modes in the MIR wavelengths that result in strong MIR light absorption. To date, researchers investigating MIR lasers for surgical applications have used bulky light sources, such as free electron lasers, nonlinear light generators, and carbon dioxide lasers. We demonstrate the use of a tiny (a few microns wide, a few millimeters long) MIR interband cascade laser (ICL) for surgical thermal ablation applications.

**Aim:** Our goal is to demonstrate the use of an ICL for surgical thermal ablation and demonstrate its efficacy in ablating normal fibroblasts and primary undifferentiated pleomorphic sarcoma tumor cells (C1619).

**Approach:** We conducted Fourier transform infrared spectroscopy analysis of healthy and cancerous tissue samples, which indicated that the absorption of tumor tissue is higher than healthy tissue around 3.3-μm wavelength. These results enabled us to select an ICL emission wavelength, λ, of 3.3  μm to probe normal fibroblast and primary undifferentiated pleomorphic sarcoma cell survival after ICL exposure.

**Results:** We show that the absorption of tumorous tissue is higher than that of healthy tissues around the 3-μm MIR wavelength. We demonstrate that the ICL is able to ablate cancer cells at very low-power levels that can be clinically implemented but that this effect does not appear to be specific to C1619 when compared to normal fibroblasts.

**Conclusions:** Our study demonstrates that ICLs may represent an exciting new avenue toward precise laser-based thermal ablation.

## 1 Introduction

Mid-infrared (MIR) light refers to wavelengths ranging from 3 to 50  μm^1^ and is the most attractive spectral region for ablation of both soft and hard tissues. This is because molecules such as water, proteins, and lipids that are contained in biological tissue exhibit molecular vibrational modes in the MIR wavelengths that result in strong MIR light absorption.^2^^–^^4^ Due to the strong MIR light absorption in tissue, substantial heating of small areas is achieved, which enables low collateral thermal damage and very precise excision of biological tissue. MIR also has a relatively shallow absorption depth of 10 to 100  μm^5^ compared to near-IR lasers currently used in laser surgeries, which penetrate the tissue at 2000 to 2500  μm.^6^ Thus, MIR lasers are not well suited to bulk tumor treatment, but may provide precision ablation after resection of the majority of the tumor by ablating to a shallow absorption depth in remaining tumor beds surrounding sensitive anatomic sites, such as around nerves. Metastatic cancers often invade microscopic, vital, and complex anatomy that is not suitable for large-scale resections favored for cancers, such as sarcomas. MIR laser technologies that can precisely damage tissue that could harbor invasive cancer cells may be of great value to patients with invasive sarcomas.

Lasers are well recognized as having promise in a variety of high-precision surgical ablation applications. They can be used to remove specific structures while preserving surrounding tissue because of their ability to focus radiation into a small area at wavelengths tuned to be selectively absorbed by a given target tissue. According to the American Cancer Society and the National Cancer Institute, the most common lasers used in ablating tumors or activating drugs are carbon dioxide (${\mathrm{CO}}_{2}$) lasers (λ=10.6  μm), argon (Ar) lasers (λ=350 to 1100 nm), and neodymium:yttrium aluminum garnet (Nd:YAG) lasers (λ=1064  nm).^7^^,^^8^ Notably, none of these common surgical lasers probe the absorption of key tissue components, such as amides and water, which have strong absorption bands around the 3- and 6-μm wavelengths. The sub-epithelium/sub-epidermis soft tissue ablation threshold energy density spectrum, shown in Fig. S1 in the Supplementary Material, indicates that around the 3- and 6-μm wavelengths, the ablation threshold is significantly reduced, which means that lasers emitting at these wavelengths are able to effectively ablate soft tissue photothermally at much lower doses than the ${\mathrm{CO}}_{2}$ lasers, which are the most common surgical lasers on the market today. A primary reason for the low ablation threshold of soft tissue at the 3- and 6-μm wavelengths is that water exhibits strong absorption at these two wavelengths.^9^ The high absorption by water at these wavelengths results in extremely short absorption depths (∼few microns) in soft tissue, which also enables precise cuts, incision, excision, and coagulation of extremely small tissue volumes with reduced collateral damage.

To date, researchers investigating MIR lasers for surgical applications have used bulky light sources such as free electron lasers; nonlinear light generators, such as chalcogenide crystal-based lasers; and ${\mathrm{CO}}_{2}$ lasers.^5^^,^^10^^,^^11^ These lasers are typically housed either in separate rooms (such as the basement of the hospital building where the operating room is located) or in large containments within the surgical room for safety reasons. The laser radiation from these other rooms or containments is brought to the surgical bed using optical fibers that experience bending and transmission losses and as a result may not provide the freedom of motion that is necessary in a surgical situation. The bulky laser systems also occupy a large footprint within the surgical room, which is not ideal.

The innovation in this work is the use of a λ∼3.3-μm interband cascade laser (ICL) that to date has not been used in biomedical applications due to lack of availability. ICLs were invented in the 1990s by Professor Rui Yang of the University of Oklahoma^12^ and are just now becoming commercially available. The advantages of ICLs include compact size (a few micrometers by a few millimeters), room-temperature operation, and high output power (∼0.5  W^13^). In this work, we demonstrate the potential use of ICLs for the development of a compact handheld laser scalpel suitable for a large variety of laser ablative applications.

The use of a λ∼3.3-μm ICL is also strategic for investigating selective ablation of tumor tissue relative to healthy tissue. Other researchers have reported^14^^–^^16^ strong absorption of tumor tissue of different cancer types as having a signature absorption around 3.03- to 3.57-μm (or 3300 to $2800\;{\mathrm{cm}}^{-1}$) wavelength band. This wavelength band is representative of stretching vibration of proteins, such as amide A and amide B, and symmetric and antisymmetric methylene ($v_{s}{\mathrm{CH}}_{2}$ and $v_{as}{\mathrm{CH}}_{2}$) stretching bands of lipids and proteins.

We first report on the Fourier transform infrared (FTIR) spectroscopy characterization of six sarcoma patient tissue samples obtained from tumors and surrounding healthy areas to study the molecular-level behavior of the tissues in the MIR wavelengths. Recently, FTIR and Raman spectroscopy approaches have been used to study cancerous specimens in MIR wavelengths, since these vibrational spectroscopic techniques allow for detecting biochemical changes in the blood and tissue samples at molecular level.^16^^,^^17^ Through our FTIR measurements, we identify several MIR wavelengths for which cancerous tissue exhibits greater absorbance than healthy tissue. Next, we report on the results obtained using a commercially available ICL with emission wavelength (λ)∼3.3  μm and 30-mW maximum output power, a relatively low-power MIR laser emitting at a wavelength where the ablation threshold is known to be minimum, to probe normal fibroblast and primary undifferentiated pleomorphic sarcoma cell survival after laser exposure. Significant cell death is seen in both groups, but preferential killing of sarcoma cells was not observed. This study demonstrates that ICLs may represent an exciting new avenue toward precise laser ablation.

## 2 Materials and Methods

### 2.1 Tissue Samples for Infrared Spectroscopy

We obtained six myxofibrosarcoma tissue samples from patients banked at the Iowa Residual Tissue Repository through an IRB-approved (ID #: 201512776) Iowa Connective Tissue Proliferative Disorder Clinical Data and Tissue Sample Collection Project. All patients signed an informed consent form before the tissues were collected post-surgery and stored as formalin-fixed paraffin-embedded tissue. Anonymity of the patient data was maintained by removing the HIPAA PHI identifiers. For each patient sample, we obtained a sample of tumor tissue as well as neighboring healthy tissue. Tissues were embedded in paraffin and then sliced to a thickness of 5  μm and mounted on silver (Ag)/tin oxide (${\mathrm{SnO}}_{2}$)-coated MIR reflective MirrIR slides (Kevley Technologies, Inc., Ohio) for FTIR measurements. MirrIR slides are recommended for FTIR-based tissue analysis, instead of the traditional glass slides used in optical microscopy analysis, because MirrIR slides have near-perfect MIR reflection and zero MIR transmission, enabling high-quality collection of MIR tissue spectral data.^18^ Corresponding tissue samples were mounted on glass slides and then stained with hematoxylin and eosin for optical microscopy examination by a trained and certified pathologist, Dr. Munir Tanas at the University of Iowa Hospitals and Clinics, to confirm the predominant (>65% of tissue) presence of either tumor or normal tissue.

### 2.2 Infrared Spectroscopy

MIR reflection spectra were collected using an FTIR Nicolet Magna 760 equipped with a mercury cadmium telluride (HgCdTe) liquid nitrogen cooled detector. Spectra were collected in the range of 4000 to $400\;{\mathrm{cm}}^{-1}$ (λ=2.5 to 25  μm) with a spectral resolution of $8\;{\mathrm{cm}}^{-1}$ and with four interferograms averaged. All spectra were collected from samples at room temperature. Figure S2 in the Supplementary Material illustrates the FTIR chamber with the sample mount and relevant optics utilized to conduct the specular reflectance measurements of the tissue samples mounted on MirrIR slides. We then used the relationship between absorption and reflection, that is, absorption=1−reflection, to obtain the absorption spectra from the measured reflection spectra.

### 2.3 Fibroblast and Cancer Cell Lines

All cell lines were cultured in humidified incubators at 37°C, 5% ${\mathrm{CO}}_{2}$, 5% and oxygen ($O_{2}$) to simulate a physiological environment. Cultures were maintained in culture media as follows: 45% DMEM (Dulbecco’s modified Eagle medium), 45% F12 nutrient mixture, and 10% fetal bovine serum (all from Gibco, Thermo Fisher Scientific, Inc.). For primary fibroblast cultures, bovine knees were obtained (Bud’s Custom Meats, Inc., Iowa), and normal bovine fibroblasts (NBF) were isolated from bovine knee synovia via collagenase/pronase digestions (0.01  mg/ml, Sigma Aldrich, Co.) in serum-free media (50% DMEM, 50% F12) overnight, followed by centrifugation and then plating onto culture flasks. Primary undifferentiated pleomorphic sarcoma tumor cells (C1619) were a generous gift of Dr. Rebecca Dodd.^19^

For laser exposures, all cells were plated into a 96-well microplate (Corning^®^ 96 Well TC-Treated Microplates size 96 wells, polystyrene, flat bottom) with one empty well between each set of test wells to decrease the risk of heating multiple wells at the same time with the laser or accidentally cross contaminating wells. The cell lines were plated separately on 96-well dishes and grown to an equal confluency prior to exposure of 80% to 90%. This high confluency was used to minimize differences in cell cycle distribution between the two cell lines, which grow at very different rates. Cell counts were confirmed using a hemocytometer.

### 2.4 Laser Exposure

A λ∼3.3-μm Fabry–Perot ICL (IF3300CM2, Thorlabs, Inc., New Jersey) was used for these studies. The representative emission spectrum and light–current curve of the ICL are shown in Fig. S3 in the Supplementary Material, which confirms that the laser emission is centered at 3.33  μm±0.01  μm and has a maximum power output of 30 mW, which corresponds to $93.75\;\mathrm{mW}/{\mathrm{cm}}^{2}$ of irradiance as defined by the $0.32\text{-}{\mathrm{cm}}^{2}$ area of the microwells in which the cells were hosted. The MIR ICL was mounted on a temperature-controlled LDMC20 laser mount from Thorlabs. The laser was operated using Thorlabs’ ITC4000 Laser Diode Current and Temperature Controller. The faceplate of the LDMC was removed during exposures to minimize the distance from the laser to the cells. The laser was thermoelectrically cooled (TEC) to 15°C using the ITC4000 temperature controller and maintained at this temperature throughout the cell line exposure experiments to maintain constant laser power across experiments.

Immediately prior to laser radiation exposure, 96-well microplates were removed from the incubator and culture media was removed from individual wells to prevent absorption by media. After ICL MIR radiation exposures, 100  μl of media was returned to each well. To determine the possible impact of 21% ambient oxygen exposure on the cells in the microplate wells during laser exposure, six control wells with media removed for 180 s but with no laser exposure were included in each microplate. Microplate wells populated with cells but with no media removal were also included.

Individual plates had three samples of each cell type for each laser exposure time. The 30-, 60-, and 90-s samples were radiated on one plate while the 180-s trials were done on a plate that was still in the incubator while the first plate was being exposed to reduce the amount of time the cells were out of the incubator. After completion of all laser exposures, 100  μl of serum-free media with no phenol red was added to each well and the dish was returned to the incubator for 1 h prior to cell viability measurements.

### 2.5 Automation of Laser Exposure

**Fig. 1:**
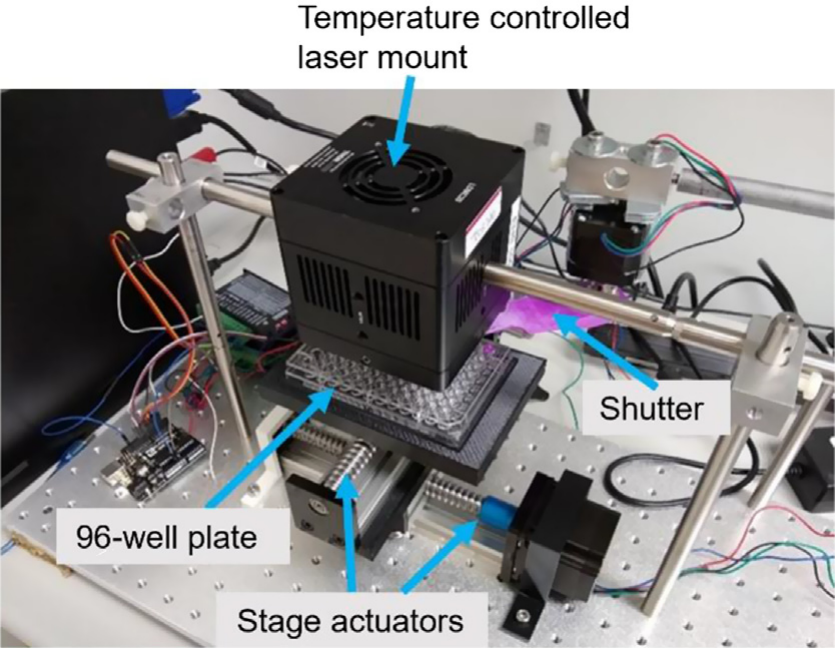
Photo showing the temperature-controlled laser mount, the 3D-printed shutter, the 96-well microplate placed on a stage, the driver motors, and the stage actuators. The opening/closing of the shutter and the precise location of the stage with respect to the laser mount were automated through a Windows workstation running a Python script.

To ensure accurate laser exposure times, we developed a computer-controlled shutter system using 3D-printed components from our lab. This system allowed us to automate the opening and closing of the shutter for precise periods of time as well as to move the stage on which the 96-well microplate was placed an exact distance with respect to the laser mount. The shutter system also allowed us to keep the ICL powered up and maintained at a constant temperature of 15°C throughout the experiments, which alleviated any variations in the ICL’s MIR light output. The shutter system consisted of two linear stage actuators controlled by two motor drivers and a third motor driver to control a shutter connected to an Arduino Uno. The setup is shown in Fig. 1. The Arduino Uno was connected via USB to a Microsoft Windows workstation during the experiment, and the shutter and stage actuators were controlled via a serial input. We wrote Python script to control and operate the shutter and the stage on which the 96-well microplate was placed. Figure S4 in the Supplementary Material shows the block diagram for the microcontroller and the motors circuitry.

The shutter was used to control ICL laser exposure times accurately. While the laser was kept on at all times and maintained at a constant temperature through the TEC controller, the shutter opened or closed for desired periods of time to expose or cover each microplate well. After a specific microplate well received the appropriate laser exposure, the stage upon which it was placed was moved precisely using the automated stage actuators.

It is important to note that the 3D-printed shutter was made of acrylonitrile butadiene styrene (ABS), which is absorbed by the 3.3-μm laser output and was effective in blocking the laser light from reaching the wells when the shutter was closed. Moreover, the 96-well plate was made of polystyrene which also absorbs 3.3-μm laser radiation; therefore, the laser output did not penetrate the cells in the adjacent wells through the sides of the wells. We confirmed the 100% absorption of the 3D-printed ABS-based shutter and polystyrene microplate at 3.3-μm MIR wavelength using the FTIR in our lab (spectra not included).

### 2.6 MTS Assay

To assess cell viability after laser exposures, we used the 3-(4,5-dimethylthiazol-2-yl)-5-(3-carboxymethoxyphenyl)-2-(4-sulfophenyl)-2H-tetrazolium (MTS) assay (Abcam plc, Massachusetts). This assay relies upon dye uptake, subsequent reduction, and retention in live cells that does not occur within dead cells. This absorbance is measured at 490 nm and conducted in the same 96-well microplate in which the cells were exposed to laser radiation. One hour after laser exposure, we added 10  μl of the MTS reagent to 100  μl of media already within the wells and then returned the plate to the incubator. A TECAN plate reader read absorbance at 490 nm. We also measured the absorbance of three wells with reagents without cells as controls. We averaged the absorbance of each set of wells exposed to a specific laser radiation dose and subtracted these values from the average of the no-cell control wells to compare the absorbance resulting from different laser doses.

### 2.7 Confocal Microscopy

For confocal microscopy images of normal and exposed cells, we plated 20,000 cells per well of a two-well chamber slide. We aspirated the porcine media from each chamber immediately before exposure, exposed the center of each chamber to a laser at 17 mW for 90 s (1.5 J of energy), and then re-added media to the chamber as rapidly as possible. One hour after exposure, we aspirated the media from all wells to remove debris, then added the viability dye calcein AM (4  μM) and the dead cell stain ethidium homodimer (2  μM) (both from Life Technologies, Thermo Fisher, Inc.) for 30 min in serum-free and phenol-red-free culture medium. Cells were then imaged with an Olympus FV1000 confocal microscope at a total magnification of 40× (4× objective), centered on the site of exposure.

## 3 Results and Discussion

### 3.1 Histopathology of Healthy and Sarcoma Tissues

Given the heterogeneity of the sarcoma cancer tissue, we tested six different patient samples to determine the absorption profile of healthy versus cancerous tissues of each sample. Table S1 in the Supplementary Material lists the patient demographics, diagnosis, tissue type, and anatomical location of the cancer. The sample set had gender diversity as well as anatomical location variation. However, the diagnosis was similar for all patients, myxofibrosarcoma.

We collected optical microscopy images of the healthy and cancerous tissue samples. The data are included in Fig. S5 in the Supplementary Material. The comparison of the optical images confirms a distinct difference between healthy and cancerous tissues as expected. Cancerous tissues clearly have the cell structure completely disrupted while it is well maintained in the neighboring healthy tissue samples. The optical microscopy analysis was primarily qualitative to confirm the structural differences in cancerous and healthy tissue samples.

**Fig. 2:**
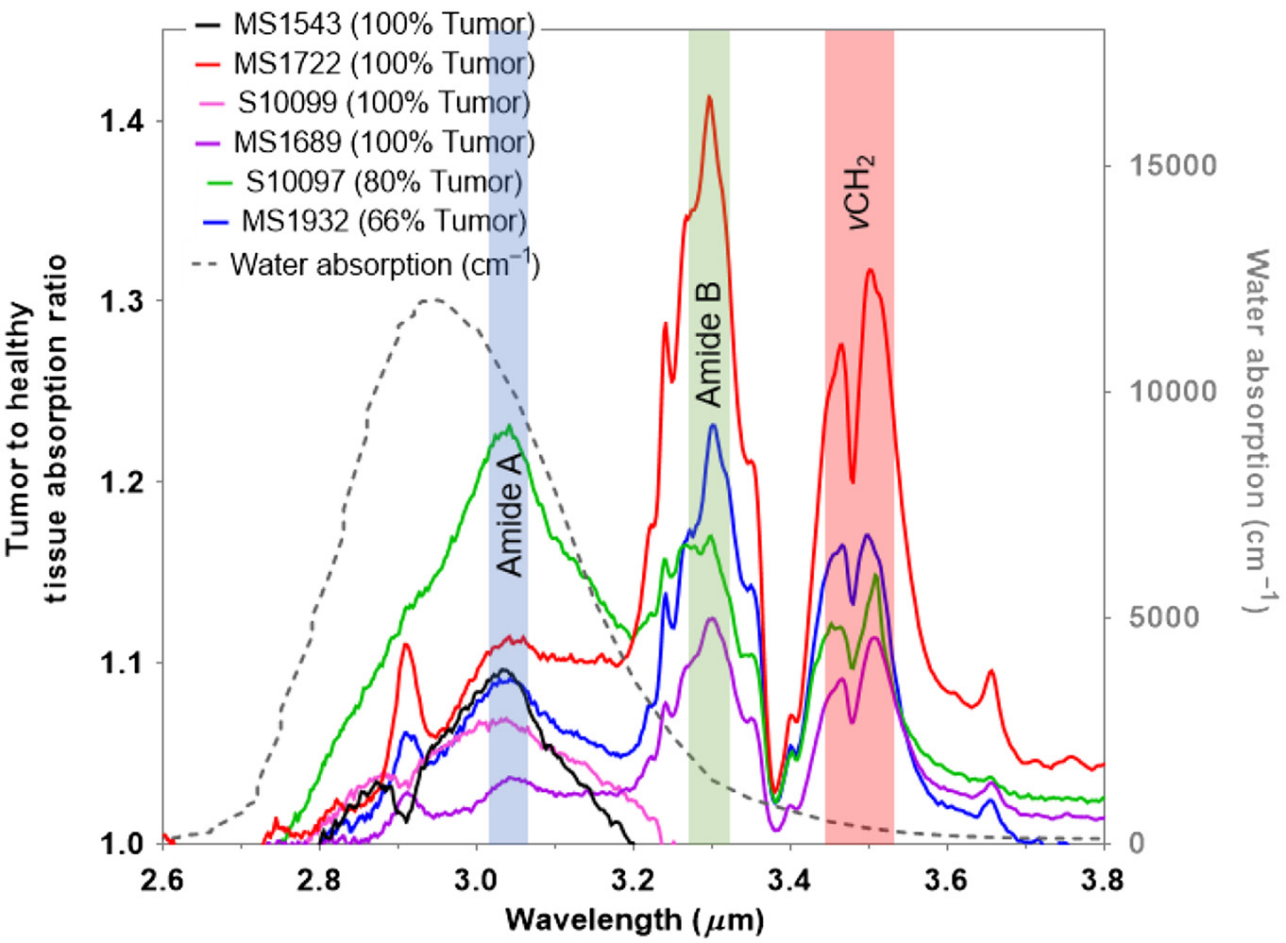
MIR spectra of tumor to healthy tissue absorption ratio (left y-axis) obtained from the analysis of FTIR measurements for the wavelengths of 2.6 to 3.8  μm. Ratio of 1 represents equal MIR absorption in tumor and healthy tissues and any ratio value larger than 1 indicates higher absorption in tumor tissue relative to healthy tissue at that specific MIR wavelength. The tissue absorption ratio spectra are overlaid on water absorption (gray dashed, right y-axis) spectrum obtained from Ref. 9. Three spectral regions are highlighted to represent the relevant stretching vibration groups of tissue proteins: amide A (blue-shaded region), amide B (green-shaded region), and methylene ($\upsilon {\mathrm{CH}}_{2}$) (red-shaded region).

Next, we conducted FTIR reflection measurements of the healthy and cancerous tissue samples mounted on MirrIR slides using the setup shown in Fig. S2 in the Supplementary Material. One MirrIR slide had just the mounting media, the IR spectra of which was also measured and subtracted from the tissue spectra. Figure 2 shows the post-processed spectral data of the tumor to healthy tissue absorption ratio as a function of wavelength. The tissue ratio spectra are overlaid on the water absorption spectra in Fig. 2 to confirm that none of the tissue spectra features are due to water absorption, which has a strong absorption band near 3-μm wavelength. Overall, Fig. 2 indicates that all six tumor tissues exhibit higher MIR absorption than their neighboring healthy tissue, as indicated by the higher than 1 absorption ratio around the 3- to 3.5-μm MIR wavelengths. Furthermore, there are three specific absorption bands that stand out in the spectral data due to their high absorption magnitude.

The first absorption band at ∼3.035  μm (or $3295\;{\mathrm{cm}}^{-1}$) is representative of the stretching vibration of amine (vNH) groups of proteins and indicates that the protein formulation is in the form of amide A, as previously shown by others.^20^^,^^21^ The second absorption band at ∼3.295  μm (or $3034.90\;{\mathrm{cm}}^{-1}$) indicates that the tissue proteins also have the configuration of amide B. In the case of amide B, the β-sheet protein structure predominates,^21^ which means that the effect of the NH group of the peptide bond ─NHCO─ is stronger than C═O, unlike in amide A, where the effect of C═O in the peptide bond is stronger. The third absorption band around 3.450 to 3.530  μm (or 2900 to $2830\;{\mathrm{cm}}^{-1}$) is representative of symmetric and antisymmetric methylene ($v_{s}{\mathrm{CH}}_{2}$ and $v_{as}{\mathrm{CH}}_{2}$) stretching bands of lipids and proteins.^22^^,^^23^ Notably, these three absorption bands have been reported to be dominant in different cancer type tissues by other researchers,^15^^,^^16^ which adds confidence in our spectral analysis.

The heterogeneity of the tissue structure for each of the patients in Fig. 2 is apparent in the MIR absorption spectra, where the absorption ratio across 2.8 to 3.6  μm varies for each sample. For example, in patient samples S10099 and MS1543, the tumor tissues exhibit higher absorption than neighboring healthy tissues, only for the amide A band. From the optical microscopy images of the tumor tissues for these two patient samples, shown in Fig. S5 in the Supplementary Material, it is evident that the cell structure is rather sparse in the tumor tissues for both cases. For patient sample S10097, tumor tissue exhibits higher absorption in the amide A band than the amide B band, while for patient samples MS1722, MS1932, and MS1689, absorption in the amide B band is higher than in the amide A band. The coexistence of both A and B protein conformations illustrates the prevalence of different hydrogen bonds that hold the protein strands together.^24^ It is known that the hydrogen bond is important in stabilizing the protein helix and that any change implies that the physiological environment has changed. Several researchers have demonstrated that these changes in hydrogen bonds are very important in characterizing disease and its progression.^20^^,^^25^^–^^27^ Finally, the FTIR spectral analysis enabled us to select an ICL emission wavelength of λ∼3.3-μm laser that would be effective in ablating tumor tissue selectively.

### 3.2 Cell Viability Analysis of ICL-Exposed Cell Lines

**Fig. 3:**
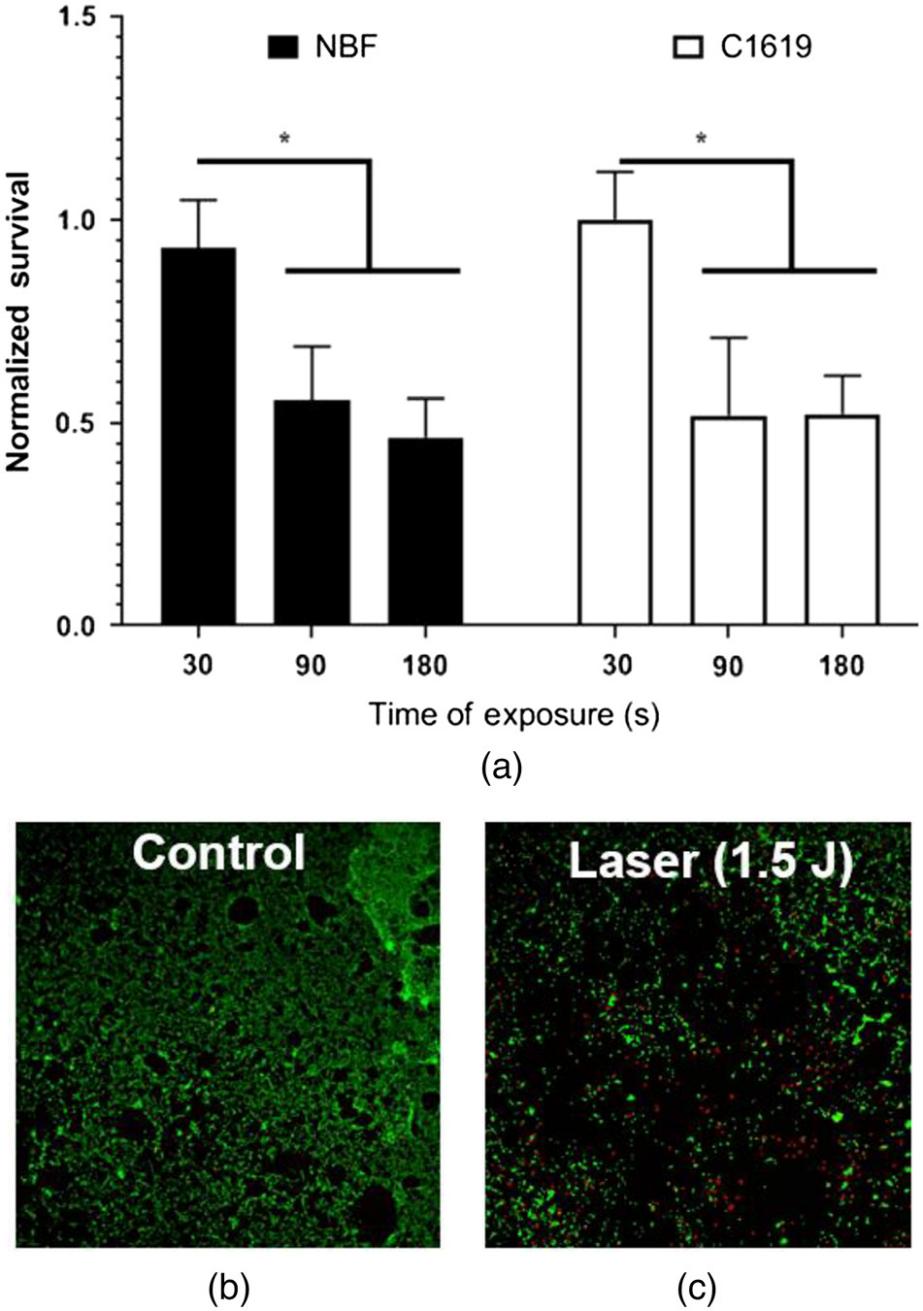
ICL causes dose-dependent cell killing in NBFs and C1619 cancer cells with no differential cell death in this model. (a) Comparison of cell death for NBF and C1619 cells after 30-mW power, 3.3-μm ICL radiation for three different exposure times, 30, 90, and 180 s. The asterisk (*) indicates the 90- and 180-s groups are different from the control with p<0.05 via two-way ANOVA. Confocal microscope images (4× magnification) of C1619 stained with (b) calcein AM live cells and (c) ethidium homodimer red dead cells. Black regions in (c) are where cell death was severe enough that cells have disintegrated or lifted off of the surface entirely. In these images, the edge of the laser spot can be clearly visualized. The size of the laser spot is likely the reason that 100% of cells in this experimental setup were not killed.

To study the duration of exposure to ICL radiation needed to kill cells cultured in a monolayer, we used the maximum power output of 30 mW from the λ∼3.3-μm ICL. With the microplate well surface area of $0.32\;{\mathrm{cm}}^{2}$, this corresponds to $93.75\;{\mathrm{mW}/\mathrm{cm}}^{2}$ of irradiance. Both the C1619 and NBF cell lines were exposed to the 3.3-μm laser radiation. Three different exposure times were chosen: 30, 90, and 180 s, which corresponds to 2.81, 8.44, and $16.87\;{J/\mathrm{cm}}^{2}$ of radiant exposure, respectively. Figure 3(a) shows the absorbance data of both cell lines using the MTS assay measured using the TECAN plate reader and normalized to control wells receiving no laser radiation. Data confirm that 30-mW laser radiation at a 3.3-μm emission wavelength can kill up to 50% of cells effectively with p<0.05 via two-way ANOVA. No statistical difference is found between the mortality rate of the two cells types after normalization.

To confirm changes in cell viability, we performed confocal microscopy on cultures of cells stained with calcein AM to indicate live cells and ethidium homodimer to indicate dead cells. Figure 3(b) shows a representative confocal microscopy image of the cell culture with no laser radiation exposure and Fig. 3(c) represents an image after exposure to 1.5 J of laser radiation. We note very little ethidium homodimer staining and an apparent lack of cellular material in Fig. 3(c). Combined with the short duration of the experiment, this suggests that cellular material had lifted off of the plates or rinsed away during staining, and implicates a necrotic cell death rather than more regulated forms of death.

These data suggest that, while FTIR identified 3.3  μm as a promising candidate, this wavelength did not provide preferential killing of cultured cancer cells over normal cells or any differential effect with MIR lasers. This lack of differential killing could be related to the culture system utilized, i.e., differences in FTIR absorbance observed may be related to tissue composition and not inherent differences of cancer cells relative to normal cells. In addition to potential differences in composition of the surrounding extracellular matrix itself, tumors also possess erratic and at times very poor blood supplies, and these differences may explain our FTIR result. Nonetheless, the power levels used in this experiment to ablate cancer and normal cells were very low and resulted in significant cell death at sites of exposure. This may prove therapeutically valuable in specific settings where shallow penetration of small areas is needed, such as within tumor beds that remain after resection around sensitive structures. Future studies may be able to refine these observations by utilizing live tissues with a mix of normal cells, tumor stroma, and tumor cells.

## 4 Conclusion

In this work, we present FTIR characterization of sarcoma and healthy tissues obtained from six patients of diverse demographic backgrounds but similar diagnoses. We demonstrate that the absorption of tumorous tissue is higher than neighboring healthy tissue around 3-μm MIR wavelengths, specifically in the amide A, amide B, and methylene absorption bands. Using this data, we then conduct studies on a cancer cell line (C1619) to study the efficacy of a relatively low-power (30 mW) ICL in ablating cell lines. We confirm cell death after ICL radiation exposure using MTS assay and confocal microscopy analysis.

## Code, Data, and Materials Availability

All relevant spectral, laser testing, and microscopy data can be made available upon request.
