# Supplementary material for: Mid-infrared absorption by soft tissue sarcoma and cell ablation utilizing a mid-infrared interband cascade laser
Source: J Biomed Opt. 2021 Apr 21;26(4):043012. doi: 10.1117/1.JBO.26.4.043012 (PMC8058894; doi:10.1117/1.JBO.26.4.043012)
Supplement: Supplementary file 1 [file JBO_026_043012_SD001.pdf]

Mid-infrared absorption by soft tissue sarcoma and cell ablation utilizing a mid-infrared interband cascade laser: supplementary information

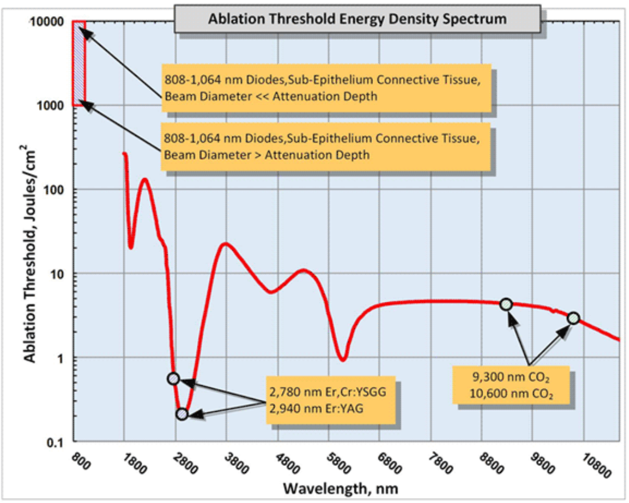

Fig. S1. Sub-epithelium/sub-epidermis soft tissue ablation threshold energy density spectrum. The data indicates that the use of λ ~3 µm interband cascade laser (ICL) could be highly energy efficient at ablating soft tissues photothermally with very low ablation thresholds. Notice that the y-axis is logarithmic. Source: "Light Scalpel: Laser Tissue Interaction" (2020), retrieved https://www.lightscalpel.com/education/surgical-co2-laser-tissue-interaction/

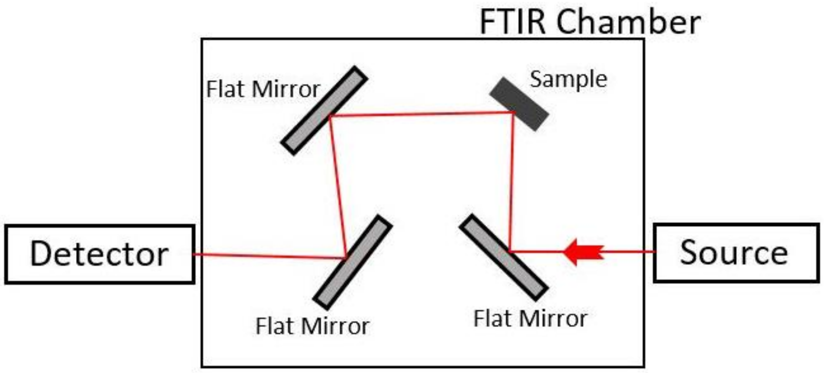

Fig. S2. Schematic of the mirror and sample arrangement within the FTIR chamber that allowed for specular reflectance measurements of the tissue samples.

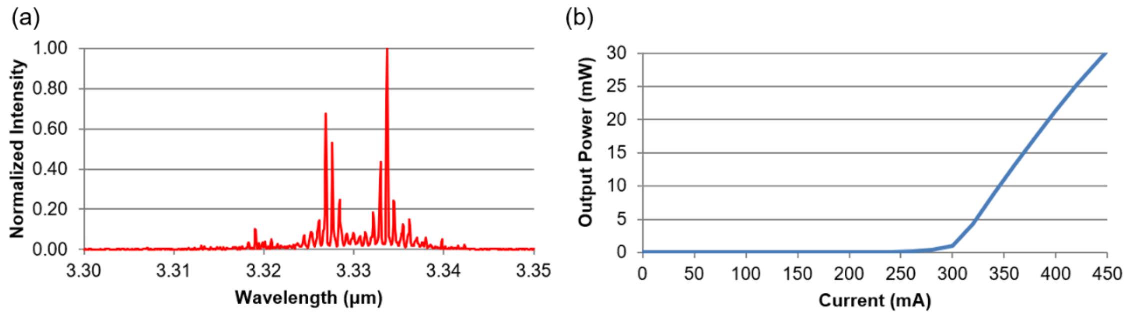

Fig. S3. Emission spectrum measured at an injection current of 448 mA (a) and light-current characteristics (b) of the λ~3.3 μm Fabry-Perot ICL (IF3300CM2, Thorlabs, Inc., New Jersey, USA) used in these studies, measured at 25°C.

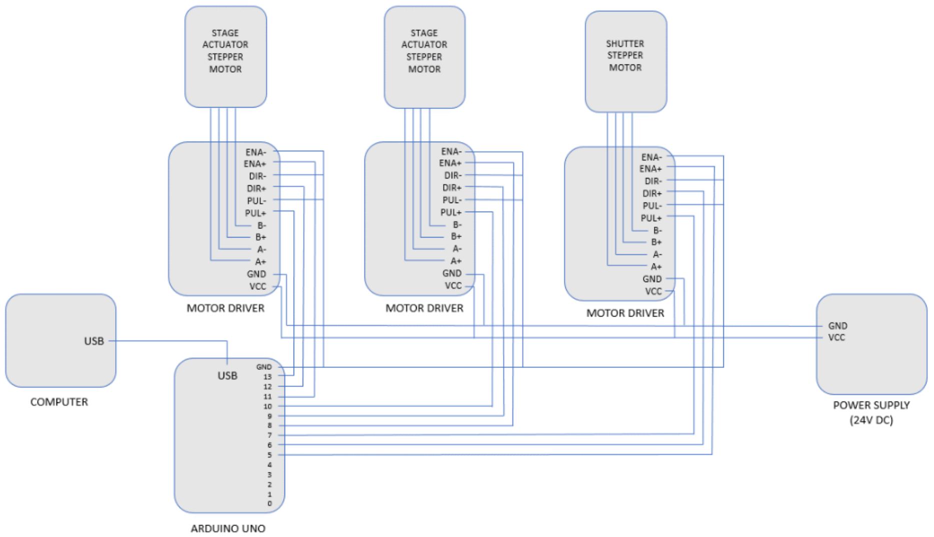

Fig. S4. Block diagram of the circuit elements and relevant electrical connections for the automated shutter and stage.

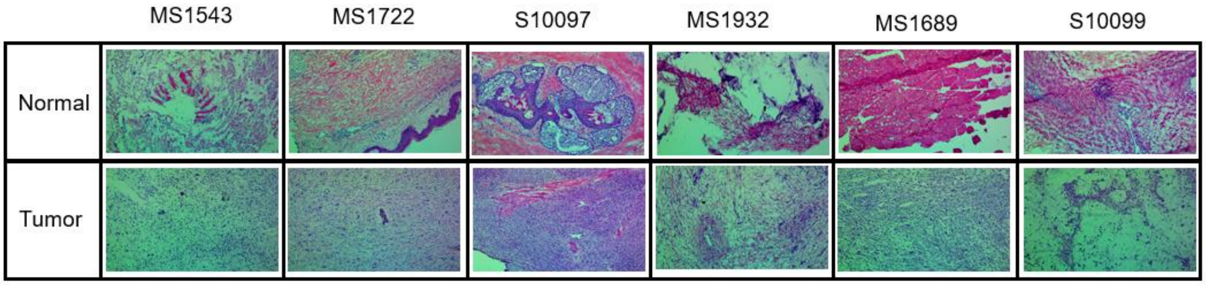

Fig. S5. Optical microscope images of the normal and tumor tissues for the six patients measured using tissues mounted on traditional glass slides. A green filter was used for the image collection, which resulted in the green background. Since the analysis of these images was to be qualitative, the use of the filter did not impact our analysis.

Subject ID	Sex	Current Mortality Status	Diagnosis	Year of Diagnosis	Age at Diagnosis	Year of Surgical Procedure	Type of Tissue	Anatomical Location	Pathological Analysis of Resected Tissue
MS1543	F	Cease to Breathe (2015)	High grade sarcoma with features of myxofibrosarcoma and pleomorphic liposarcoma	2014	58	2014	Soft	Hip & thigh (upper leg)	100% tumor
MS1722	F	Alive	Myxofibrosarcoma	2015	63	2015	Soft	Elbow & forearm	100% tumor
S10097	M	Alive	Dermatofibrosarcoma protuberans	2016	47	2017	Soft	Shoulder	80% tumor
MS1932	M	Alive	Myxofibrosarcoma	2016	66	2016	Soft	Leg-Lower (shin, calf)	66% tumor
MS1689	M	Alive	Myxofibrosarcoma	2015	62	2015	Soft	Leg-Upper, thigh	100% tumor
S10099	M	Alive	Myxofibrosarcoma	2017	48	2017	Soft	Elbow & forearm	100% tumor

Table S1. Patient demographics, diagnosis, type of tissue, and anatomical location included in this study.
